# Process, outcomes and feasibility of implementing universal newborn screening programme in Sri Lanka

**DOI:** 10.1186/s12887-026-07113-w

**Published:** 2026-06-05

**Authors:** Sachith Mettananda, Nethmini Thenuwara, Ranod Madushith, Yasara Samarakoon, Nishani Lucas, Himali Herath, Manjula Danansuriya

**Affiliations:** 1 Department of Paediatrics, Faculty of Medicine – University of Kelaniya, Thalagolla Road, Kelaniya, Ragama, 11010 Sri Lanka; 2 Colombo North Teaching Hospital, University Paediatrics Unit, Ragama, Sri Lanka; 3 Family Health Bureau, Colombo, Sri Lanka; 4 World Health Organisation Country Office, Colombo, Sri Lanka; 5 Department of Paediatrics, University of Colombo, Colombo, Sri Lanka; 6 Neonatal Intensive Care Unit, De Soysa Hospital for Women, Colombo, Sri Lanka

**Keywords:** Newborn screening, Eye abnormalities, Congenital hypothyroidism, Congenital heart diseases, Congenital deafness, Sri Lanka

## Abstract

**Background:**

The universal newborn screening detects clinically occult but potentially serious conditions, enabling timely treatment to reduce child morbidity and mortality. The World Health Organisation recommends incorporating universal newborn screening into routine newborn care. In early 2025, Sri Lanka launched a universal newborn screening package including screening for eye abnormalities, congenital hypothyroidism, critical congenital heart diseases and hearing impairment in five pilot hospitals. Here, we aim to describe the process, outcomes, and technical and operational feasibility of implementing the universal newborn screening package in Sri Lanka.

**Methods:**

A descriptive study was conducted in five hospitals that piloted the universal newborn screening package in Sri Lanka from January to March 2025. All neonates born during the study period were recruited. Data on the screening process were obtained from maternal interviews and patient records. Telephone interviews were done 4–6 weeks post-discharge to capture screening outcomes. Neonates who had abnormal results of the screening tests were identified as ‘Refer’ for the test.

**Results:**

A total of 3860 newborns (males-51.0%) were recruited. Eye screening was performed in > 99% of neonates, with 76 (1.9%) being ‘Refer’ predominantly (74/76) due to the presence of risk factors. The screening rate for congenital hypothyroidism was 99.8%, which detected six (0.15%) with congenital hypothyroidism. Pulse oximetry screening was performed in 98.5%, of which eight (0.2%) were ‘Refer’ and one subsequently confirmed to have a critical congenital heart disease. Overall, 79.1% of neonates underwent hearing screening, of which 325 (10.6%) were ‘Refer’ in the first test, and 20 were ‘Refer’ in the second test and referred for further evaluation.

**Conclusions:**

The pilot implementation of the Universal Newborn Screening package in Sri Lanka demonstrated high coverage exceeding 98% for screening of congenital hypothyroidism, critical congenital heart disease, and eye abnormalities, highlighting operational feasibility. In contrast, screening for hearing impairment remained suboptimal, largely attributable to logistical and system-level challenges. The congenital hypothyroidism screening detected six (0.15%) cases, and pulse oximetry screening detected one with a critical congenital heart disease. These findings suggest that universal newborn screening is a feasible and potentially sustainable intervention that can be effectively integrated into the existing health system in Sri Lanka.

## Introduction

Newborn screening is a critical component of maternal and child health in a country. It is defined as point-of-care examinations and laboratory evaluations performed on newborn infants to identify clinically occult but potentially serious disorders that require expedient interventions [1]. Since their inception, newborn screening programmes have helped to reduce neonatal, infant, and under-5 mortality worldwide [2].

The World Health Organisation (WHO) recently recommended incorporating universal newborn screening as part of discharge readiness and preparedness for routine newborn care [1]. In 2022, the WHO South East Asia Regional Office (SEARO) released implementation guidelines on universal newborn screening for hearing impairment, eye abnormalities, and neonatal hyperbilirubinemia before discharge to support countries in adopting the WHO Newborn Screening Recommendations [3]. Early identification of these conditions allows timely interventions to prevent severe health issues and improve long-term outcomes [4].

Birth defects remain a significant public health concern in Sri Lanka, ranking among the leading causes of both neonatal and infant mortality, accounting for approximately 39% of infant deaths [5]. This underscores the need for strengthened preventive and early detection strategies, especially to achieve the targets of the Sustainable Development Goals.

Newborn screening in Sri Lanka has been implemented for decades. However, it was initially limited to a complete neonatal examination by a trained doctor before discharge [6]. In 2016, universal screening for congenital hypothyroidism using heel prick blood sampling for thyroid-stimulating hormone (TSH) was introduced [7, 8]. The newborn screening program was expanded in 2018 to include pulse oximetry screening for critical congenital heart disease (CCHD) [9]. Although newborn screening for congenital deafness using the otoacoustic emission (OAE) test was introduced in Sri Lanka in 2019, it had not been scaled up due to limited resources.

In early 2025, the Ministry of Health of Sri Lanka, with technical guidance from WHO, launched an initiative to pilot implement the Universal Newborn Screening (UNS) as a package at five selected teaching hospitals. The new universal newborn screening package included screening for eye abnormalities, congenital hypothyroidism, CCHD and hearing impairment. Here, we aim to describe the process, outcomes, and technical and operational feasibility of implementing the universal newborn screening package in Sri Lanka.

## Methods

A descriptive study was conducted in five public-sector tertiary care hospitals in Sri Lanka, which were pilot implementing the UNS package. The participating hospitals, Castle Street Hospital for Women (Colombo, Western Province), De Soysa Hospital for Women (Colombo, Western Province), Sri Lanka-German Friendship Teaching Hospital (Galle, Southern Province), Teaching Hospital Kurunegala (North Western Province), and Teaching Hospital Jaffna (Northern Province) were distributed across four out of nine provinces of Sri Lanka. These hospitals represented different geographical regions, service contexts and high-volume delivery facilities in the country.

All live-born neonates delivered and subsequently discharged from these hospitals between January and March 2025 were eligible for recruitment. All neonates born in the study hospitals were included, while stillbirths and neonates transferred out before discharge were excluded. The 3-month study period was chosen based on feasibility, logistical and funding considerations.

The UNS package consisted of screening for eye abnormalities, congenital hypothyroidism, CCHD, and hearing impairment. Screening for eye abnormalities included assessment of risk factors for visual impairment (prematurity, hypoxic injury at birth, family history of visual impairment, structural facial defects, syndromes, hydrocephalus, and evidence of congenital infections), external eye examination, and evaluation of the red reflex. The red reflex was evaluated by an experienced, trained medical officer using an ophthalmoscope at a dark corner of the ward. Presence of risk factors, any abnormalities in the external eye examination or absent, dull, or asymmetrical red reflex were classified as ‘Refer’. Those who were ‘Refer’ were referred to a consultant ophthalmologist in the same hospital.

For congenital hypothyroidism, dried blood spots were collected via heel prick blood sampling by a nurse before discharge and analysed in a clinically accredited laboratory for TSH levels using Fluoroimmunoassay (FIA). Neonates with TSH >6mIU/L were considered ‘Refer’ and referred to a consultant paediatrician or neonatologist in the same hospital for further management and follow-up [10].

Screening for CCHD was performed using pulse oximetry by a nurse on the second day of life, measuring pre-ductal (right hand) and post-ductal (any foot) oxygen saturation. Saturation < 95% in either site or pre- and post-ductal saturation difference > 3% was categorised ‘Refer’. Those who were ‘Refer’ in CCHD screening were referred to a consultant paediatric cardiologist for urgent echocardiography performed on the same day.

Hearing screening was performed between 24 and 72 h after birth using OAE testing by a trained nurse or an audiologist. Neonates who failed in the OAE assessments were classified as ‘Refer’ and were given an appointment for the second OAE test after 2–4 weeks. Those who were ‘Refer’ in the second OAE were referred to an otolaryngology specialist in the same hospital.

For all screening tests, neonates with normal results were categorised as ‘Pass’ and those with abnormal findings as ‘Refer’ to minimise stigma attached. The results of all screening tests, except for congenital hypothyroidism screening, were communicated to the mother by the staff member performing the screening immediately after the test. The TSH results were communicated directly to the mothers after discharge, either via mobile phone text messages or by public health midwives during home visits within two weeks.

Data collection was conducted in two phases using structured instruments by dedicated data collectors. In the hospital-based phase, trained data collectors visited each hospital daily and recruited all neonates discharged on that day. Data were obtained from maternal interviews, hospital records, and the Child Health Development Record (CHDR), which included demographic details, timing and procedures of screening, and test results. In the post-discharge follow-up phase, mothers of all recruited neonates were contacted by telephone 4–6 weeks after discharge. Using a pretested interviewer-administered questionnaire, information was obtained on postnatal complications, results of confirmatory or follow-up testing, and any interventions initiated for neonates with ‘Refer’ results.

Before study initiation, data collectors received standardised training on screening procedures, data recording, and ethical conduct. The training aimed to ensure data quality across study sites. Periodic supervisory visits were conducted, and daily verification of data forms and random cross-checks were carried out to maintain consistency and accuracy.

All data were entered into a centrally-regulated, password-protected database using a Google Form^®^ and checked for completeness and consistency before analysis. Statistical analyses were conducted using IBM SPSS Statistics version 29.0. Descriptive statistics, including frequencies, percentages, means, and standard deviations, were used to summarise demographic characteristics and screening outcomes.

Ethical approval for the study was obtained from the Ethics Review Committee of the Sri Lanka College of Paediatricians (Ref. No. SLCP/ERC/2024/15). Written informed consent was obtained from all mothers prior to recruitment, and administrative approval was obtained from the Director General of Health Services and the Directors of all participating hospitals. Participant confidentiality was maintained by anonymising data before analysis.

## Results

A total of 3860 newborns were recruited between January and March 2025, of which 1969 (51.0%) were boys, and 1886 (48.9%) were girls. Most (3737, 96.8%) were born following singleton pregnancies, while 123 (3.2%) babies were born following twin or higher-order pregnancies. Most neonates (2070, 53.6%) were delivered by vaginal delivery, and 1790 (46.4%) were delivered by caesarean section. The study population represented all ethnic groups and socio-demographic classes of Sri Lanka (Table 1).

**Table 1:** Basic and socio-demographic characteristics of the study population

Characteristic	Frequency (*N* = 3860)	Percentage
Hospital		
Castle Street Hospital for Women, Colombo	783	20.3%
De Soysa Hospital for Women, Colombo	665	17.2%
Sri Lanka German Friendship Teaching Hospital, Galle	1159	30.0%
Kurunegala Teaching Hospital	827	21.4%
Jaffna Teaching Hospital	426	11.0%
Sex		
Male	1969	51.0%
Female	1886	48.9%
Undetermined	5	0.1%
Ethnicity		
Sinhala	2561	66.3%
Tamil	611	15.9%
Muslim	612	15.9%
Other	75	2.0%
Marital status		
Married	3821	99.0%
Unmarried	31	0.8%
Divorced / Separated	5	0.1%
Widowed	3	0.1%
Mother’s employment status		
Housewife	2745	71.1%
Working mother	1115	28.9%
Mother’s education level		
No schooling	11	0.3%
Only primary education	255	6.6%
Up to Ordinary Level (Grade 11)	1444	37.4%
Up to Advanced Level (Grade 13)	1398	36.2%
Diploma	226	5.9%
Degree	526	13.6%
Monthly family income (LKR)*		
=<20,000	127	3.3%
20,001–50,000	1398	36.5%
50,001-100,000	1767	46.1%
100,001-300,000	501	13.1%
> 300,000	40	1.0%

* USD 1 ~ LKR 300

One hundred eighty-two (4.7%) neonates were born to consanguineous parents. Family history of congenital heart diseases, congenital hearing impairment and congenital hypothyroidism were reported by 45 (1.2%), 29 (0.8%) and 8 (0.2%) mothers, respectively.

The follow-up data collection was conducted 4–6 weeks after discharge by contacting mothers by telephone, with repeated attempts if initial calls were missed. Data on 3183 newborns were collected during the follow-up, yielding a response rate of 82.4%.

### Screening for eye abnormalities

Seventy-four (1.9%) newborns had risk factors for eye abnormalities, of which prematurity was the most common (Table 2).

**Table 2:** Frequency of occurrence of risk factors for eye abnormalities

Risk factor	Frequency (%) (*N* = 3860)
Prematurity (< 32 weeks)	31 (0.80%)
APGAR < 7 at 1, 5 or 10 min (Hypoxic injury at birth)	25 (0.65%)
Family history of blindness or visual impairment (e.g., congenital cataracts, congenital glaucoma, retinoblastoma)	9 (0.23%)
Structural birth defects in facial structures	5 (0.12%)
Syndromes (e.g., Down syndrome)	4 (0.10%)
Hydrocephalus	1 (0.02%)
Evidence of congenital infections (e.g., cytomegalovirus, herpes, rubella, syphilis and toxoplasmosis)	1 (0.02%)

**Fig. 1:**
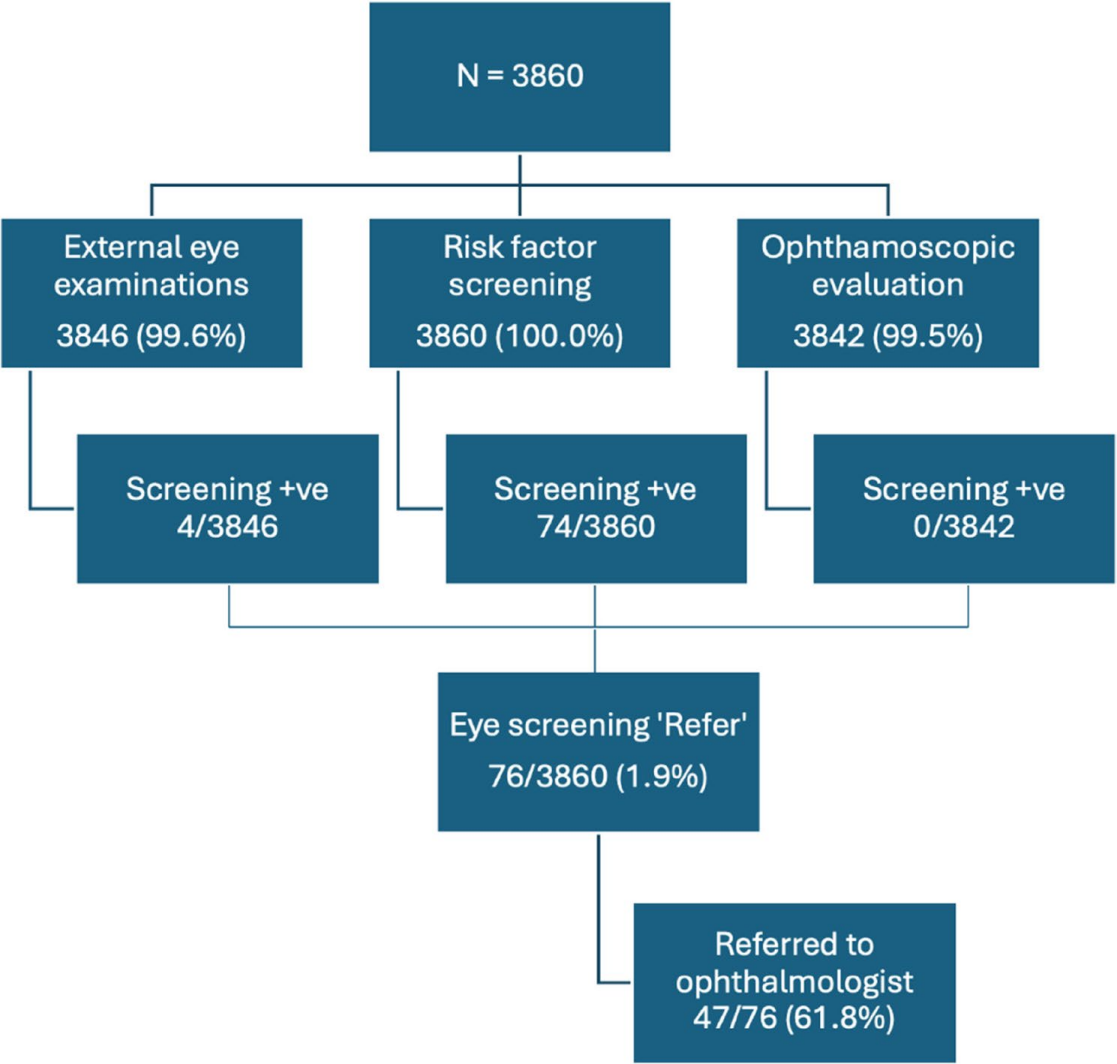
Care pathway and outcomes of screening for eye abnormalities

External eye examination was performed in 3846 (99.6%) newborns, and the ophthalmoscopy examination was done in 3842 (99.5%) (Fig. 1). The results of the screening for eye abnormalities were ‘Refer’ in 76 (1.9%) neonates due to the presence of risk factors (*n* = 74), eyelid haemangioma (*n* = 1), squint (*n* = 1) and eye discharge (*n* = 2). Only 47/76 (61.8%) neonates who were ‘Refer’ in eye screening were referred to ophthalmologists.

### Heel prick TSH screening for congenital hypothyroidism

**Fig. 2:**
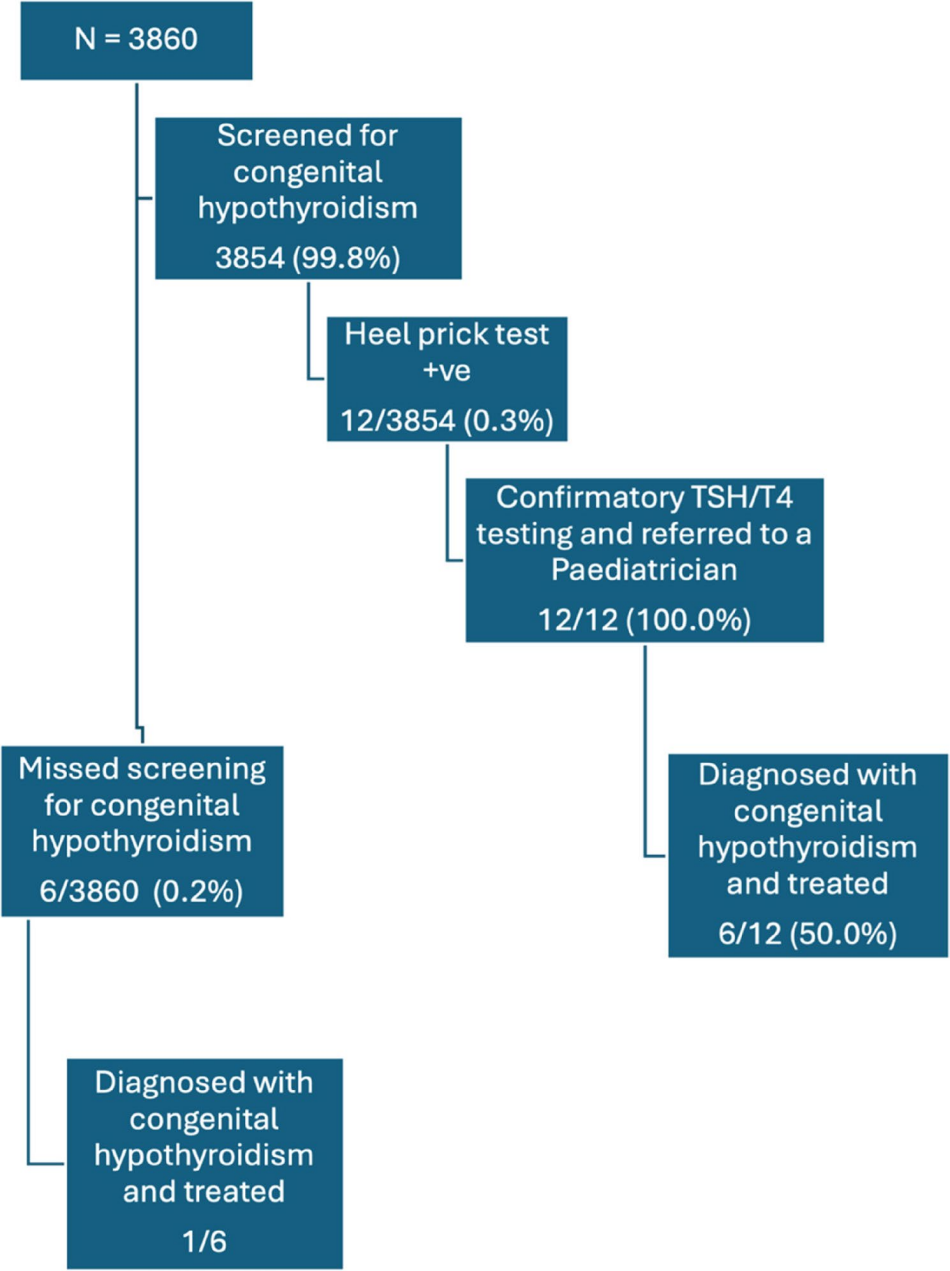
Care pathway and outcomes of screening for congenital hypothyroidism

The screening for congenital hypothyroidism by heel-prick blood sampling for TSH was performed in 3854 newborns, yielding a screening rate of 99.8%. Twelve neonates had abnormal initial heel-prick TSH reports yielding a screen positive rate of 0.34% (Fig. 2). Following confirmatory investigations, 6/12 were diagnosed with congenital hypothyroidism and the remaining 6/12 were false positives; therefore, the positive predictive value of heal prick TSH test was 50%. All neonates diagnosed with congenital hypothyroidism were started on levothyroxine treatment at a median age of 15.5 (range: 3 to 33) days. In addition, the follow-up data collection identified one patient who had missed the hypothyroidism screening and was diagnosed with congenital hypothyroidism at 30 days by investigations done on clinical suspicion. Including the missed case, the prevalence of congenital hypothyroidism reported in our cohort was 1.8 per 1000 newborns (7/3860).

### Pulse oximeter screening for critical congenital heart diseases

**Fig. 3:**
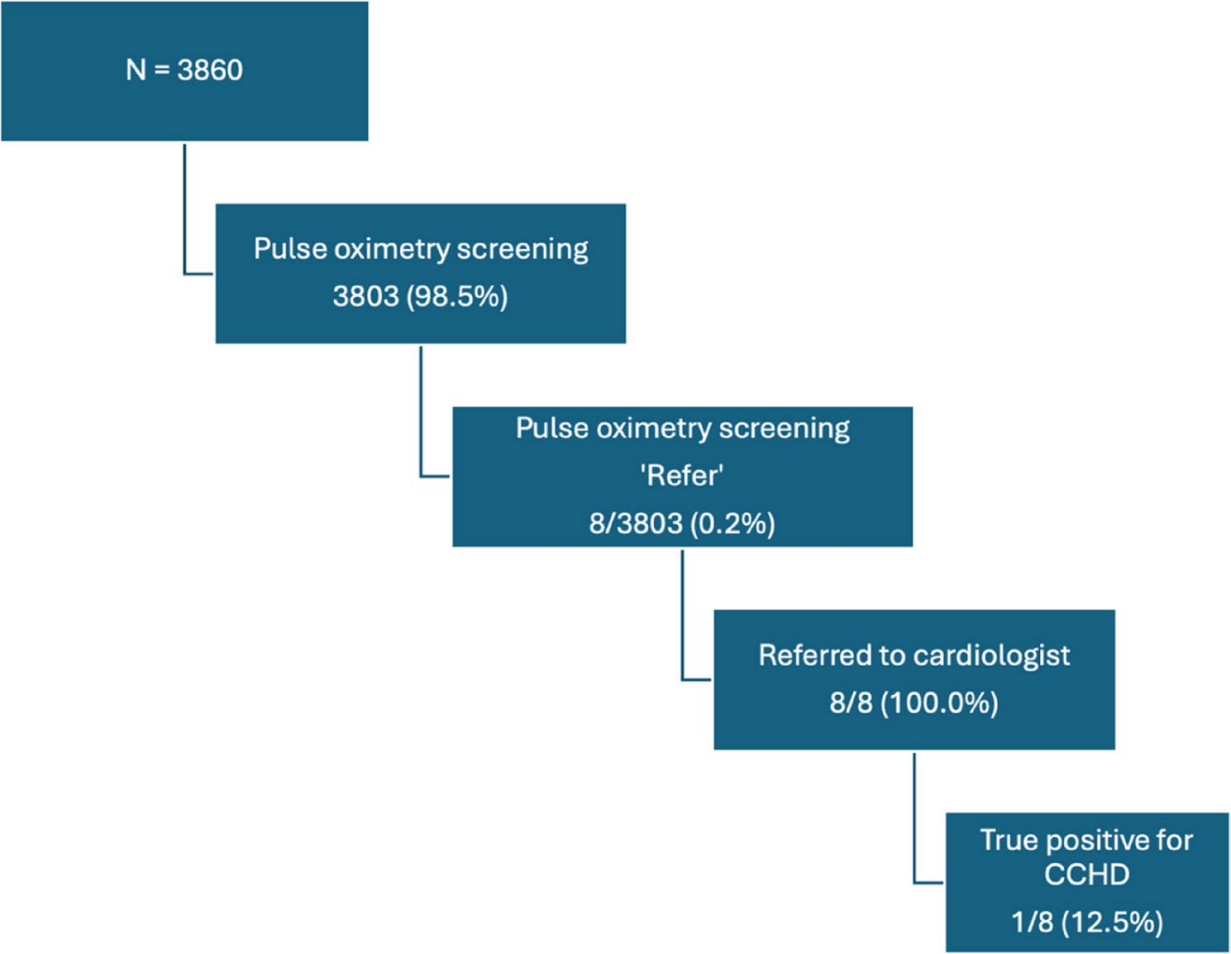
Care pathway and outcomes of screening for critical congenital heart diseases

The screening for CCHD by pulse oximetry was performed in 3803 (98.5%) neonates (Fig. 3). Eight (0.2%) neonates were ‘Refer’ in the pulse oximetry screening and were referred to the cardiologists for urgent echocardiogram on the same day. Echocardiography confirmed a CCHD - a complex cyanotic heart disease - in one neonate (Table 3). The follow-up data collection did not reveal any newborns missed in the pulse oximetry screening and later diagnosed with a CCHD.

**Table 3:** Echocardiography diagnoses of newborns who were screened positive on pulse oximetry

No.	Echocardiography diagnoses
1	Complex cyanotic congenital heart disease. Truncus arteriosus type 1, thickened doming four-leaflet truncal valve with valve stenosis and regurgitation, large conoventricular ventricular septal defect, bilateral superior vena cava, small ostium secundum atrial septal defect, significant flow reversal of descending aorta
2	Mild to moderate pulmonary hypertension
3	Small patent foramen ovale, trivial aortic regurgitation, tiny patent ductus arteriosus, moderate to severe pulmonary hypertension
4	Small patent foramen ovale, ostium secundum atrial septal defect, tiny patent ductus arteriosus
5	Large ventricular septal defect, mild pulmonary stenosis, moderate-sized ostium secundum atrial septal defect
6	Small ostium secundum atrial septal defect, mild septal hypertrophy, persistent pulmonary hypertension of the newborn
7	Moderate ostium secundum atrial septal defect, moderate persistent pulmonary hypertension of the newborn, mild right ventricular dilatation
8	Ostium secundum atrial septal defect, moderate persistent pulmonary hypertension of the newborn

### Screening for hearing impairment

Risk factors for hearing impairment were identified in 175 (4.5%) neonates. The most common risk factor was gentamicin use in the mother or baby (Table 4). Although all neonates with risk factors were supposed to be referred to an otolaryngologist, only 58/175 (33.1%) had been referred to an otolaryngology specialist. The likely reason for the low referral rate is the very short duration of gentamicin treatment deemed safe by treating clinicians.

**Table 4:** Frequency of occurrence of risk factors for hearing impairment

Risk factor	Frequency (%) (*N* = 3860)
Ototoxic medication (e.g., gentamicin, furosemide, cisplatin) use in the mother or baby	71 (1.83%)
Meningitis or sepsis	65 (1.68%)
Admission to a neonatal intensive care unit for > 48 h	51 (1.32%)
Prematurity (< 34 weeks)	33 (0.85%)
Family history of hearing loss in childhood	15 (0.38%)
Hypoxic ischemic encephalopathy grade 2 or 3	2 (0.05%)
Craniofacial malformations or syndromes	4 (0.10%)
Bilirubin levels at or above exchange levels	2 (0.05%)
Maternal history suggestive of congenital infections (e.g., cytomegalovirus, herpes, rubella, syphilis and toxoplasmosis)	1 (0.02%)

**Fig. 4:**
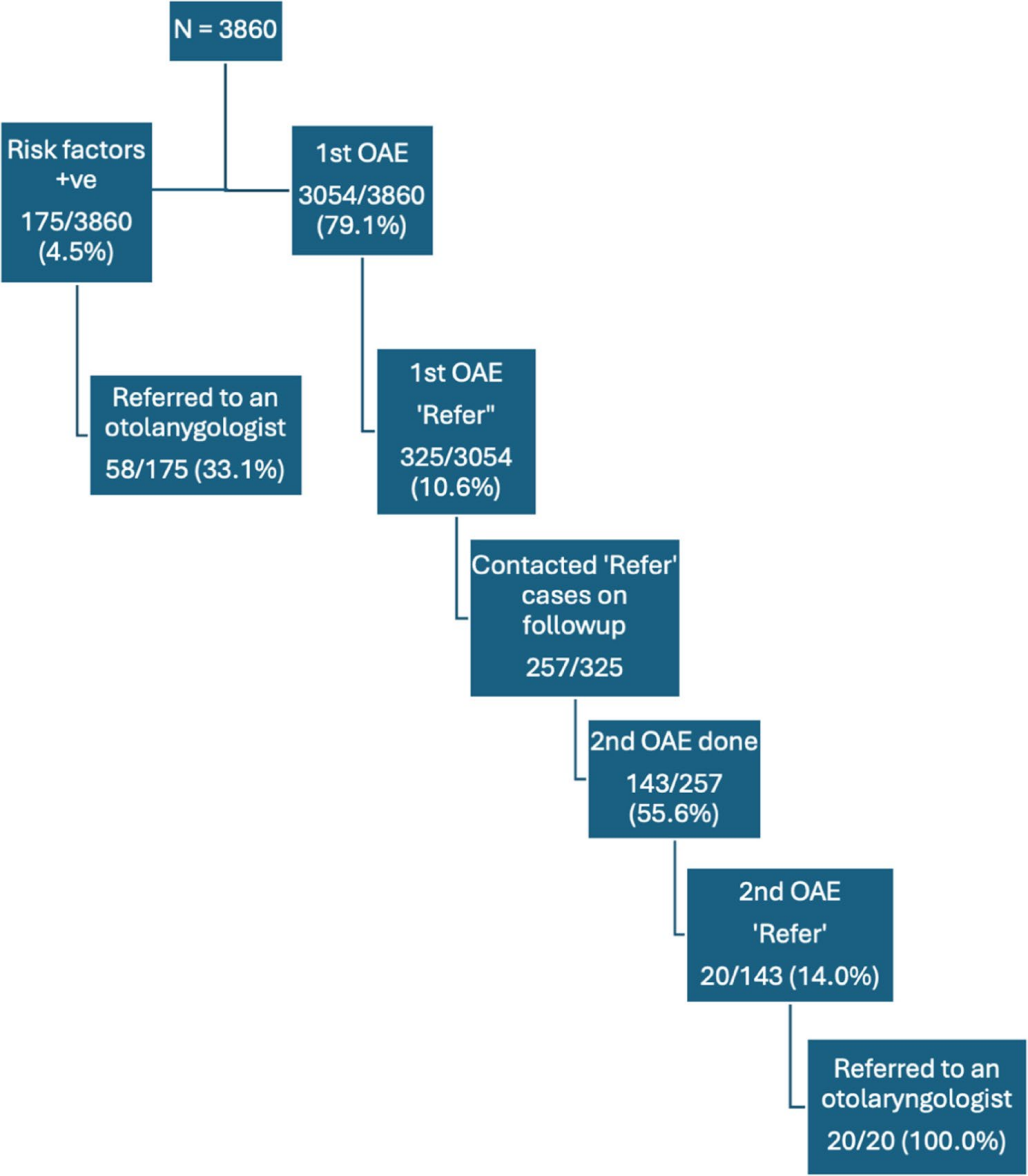
Care pathway and outcomes of screening for hearing impairment

Overall, 3054 neonates underwent OAE, achieving a hearing screening rate of 79.1% (Fig. 4). Recurrent malfunctions of OAE machines, limited equipment availability relative to birth volume, and unavailability of trained staff were the most reported reasons for not performing hearing screening in others. Of the neonates who underwent hearing screening, 325 (10.6%) were ‘Refer’ in the first OAE test; 215 (7.0%) were ‘Refer’ in both ears, 56 (1.8%) were ‘Refer’ only in the left ear, and 54 (1.8%) were ‘Refer’ only in the right ear. All those who ‘Refer’ in the first OAE were given appointments for the second OAE within 2–4 weeks.

Of the 325 neonates who were ‘Refer’ in the first OAE test, only 257 (79.1%) could be contacted post-discharge despite repeated attempts. Of the 257 who were contactable, only 143 (55.6%) had attended the second OAE test. Among them, 123 (86%) were ‘Pass’ and 20 (14.0%) were ‘Refer’ in the second OAE; 11 (8%) were ‘Refer’ in both ears, 2 (1%) were ‘Refer’ only in the left ear, and 7 (5%) were ‘Refer’ only in the right ear. All neonates who were ‘Refer’ in the second OAE have been referred to otolaryngology specialists. All of them were on otolaryngology follow-up with either a plan for repeat hearing test (n = 15) or referral to paediatric otolaryngologists (n = 1) by the end of the data collection period.

## Discussion

This study demonstrated that screening coverage for three of the four components of the UNS package was high across all five hospitals, with screening rates exceeding 98%. Screening for eye abnormalities and congenital hypothyroidism achieved coverage rates above 99%, while CCHD screening achieved a rate of 98.5%. These results were achieved using the existing health staff without deploying additional human resources. The research team’s data collectors were involved only in gathering data, following up newborns along referral pathways to collect outputs, and conducting telephone interviews with mothers during the post-discharge period to collect outcomes. These findings indicate high feasibility and broad acceptance of the UNS package within routine public sector perinatal services. The coverage observed in this initiative is considerably higher than the screening rates reported in other low- and middle-income countries, suggesting that the proposed package can be effectively integrated into Sri Lanka’s existing maternal and child health programme [11, 12].

Sri Lanka has a state-run, tax-funded health care system that provides both preventive and curative treatment free of charge to all citizens [13]. The free services cover all aspects of care, including antenatal care, in-hospital delivery, neonatal care, newborn screening, follow-up of screening-positive neonates, as well as any treatment required, including corrective surgery for complex congenital heart diseases and cochlear implants for congenital deafness. All five hospitals selected for the study are state hospitals and have the facilities to provide tertiary-level care to all screen-positive neonates at no cost.

Eye examination had been part of routine neonatal examination for decades; however, the said UNS package introduced dedicated orientation for health staff, structured protocols with a more specific, detailed algorithm for evaluating those at high risk of eye abnormalities, separate risk-factor screening, and clear referral pathways. The UNS package also focused on data recording and data entry tools in the ward setting. We observed that although screening rates for eye abnormalities exceeded 99%, referral rates for neonates with risk factors for eye abnormalities were only 61.8%, indicating room for improvement. The low referral rate is most likely due to the screening algorithm still being in the pilot stage and not yet fully integrated into the neonatal examination checklists and the CHDR of Sri Lanka. Strengthening the integration of this algorithm into routine workflows, staff training and consistent documentation are essential to improve referral adherence.

The screening coverage for congenital hypothyroidism in this study was nearly universal, consistent with the high uptake already achieved since Sri Lanka introduced national newborn screening for congenital hypothyroidism in 2016 [11]. The incidence rate observed in this cohort, 1.8 per 1000 live births, was higher than the globally reported range of 1:2000 to 1:4000 and the rates reported from other South Asian countries [12, 14]. It was also marginally higher than the previously reported national incidence of 1 in 1266 from a 2019 study conducted in Sri Lanka [15]. These findings could reflect improvements in case detection, regional variations, or methodological differences in screening coverage and case confirmation. Further population-based research is warranted to explore these trends.

Pulse oximetry screening for CCHD identified eight ‘Refer’ newborns (0.2%) with positive results who were subsequently referred for echocardiography, of which one infant was confirmed to have CCHD. No additional CCHD cases were identified during post-discharge follow-up, indicating that the screening algorithm had an acceptable sensitivity for detecting CCHD in this population. The detection rates observed were comparable to those reported in high-income settings [16, 17].

The screening rate for hearing impairment (79.1%) was notably lower than for other components of newborn screening and the widely accepted screening rate benchmark of > 95% [18]. The hearing impairment screening rate was lower than that in other pilot studies conducted in the region [19]. Several operational challenges, including recurrent malfunctions of OAE machines, limited equipment availability relative to birth volume, sharing equipment between two or more units with high birth rates, and inadequate numbers of trained personnel to perform screenings due to leave or staff transfers, contributed to the lower coverage. OAE testing is time-consuming, and the timing of the test overlapped across units that shared the same machine. As hearing screening was newly introduced in Sri Lanka, staff members had limited experience in troubleshooting minor technical issues, despite having received hands-on training prior to programme implementation. Addressing these logistical and capacity constraints will be critical for achieving full-scale integration of hearing screening into the national UNS programme.

Another important observation was that 114 (44.3%) of 257 neonates who were “Refer” in the first OAE and were contactable by telephone had not attended the second OAE. The likely reasons for this non-compliance were the practical difficulties of attending follow-up for the neonate after discharge, parents’ perception that their baby has normal hearing despite being ‘Refer’ on the first OAE, and miscommunication during the second OAE test at discharge. Strengthening post-test counselling after newborn screening and parent education activities on UNS could help to improve the utilisation of freely available care pathways.

One limitation of our study was that it was limited to five selected tertiary care hospitals, compromising its generalisability to Sri Lanka. Recent data indicate that 41% of all newborns in the country are delivered in Teaching Hospitals, whereas the remaining 59% are delivered in lower-tier Base, District General and Divisional Hospitals [20]. Teaching Hospitals provide specialised management with three or more obstetric units led by consultants and a level 3 neonatal intensive care, whereas Base and District General Hospitals have fewer staff and specialists and have only one or two obstetric units. Therefore, lower-tier hospitals in the country will not have the same level of facilities and expertise for newborn screening and implementing the UNS programme in these hospitals could be challenging.

Relatively low response rate (82.4%) for the telephone follow-up is another limitation of our study. Providing incorrect mobile phone numbers, mobile phone number updates, and refusal to provide information over the telephone due to privacy concerns were the reasons for the relatively low follow-up response rate. The loss to follow-up cohort could have led to missed false negatives of screening and introduced a bias when determining the sensitivity of screening tests. This was mainly a limitation in the interpretation of hearing impairment screening, as 68 out of 325 (20.9%) newborns who were ‘Refer’ in the first OAE were not contactable.

While newborn screening has been a longstanding practice in Sri Lanka, this study introduces a significant advancement. Guided by recent WHO recommendations, we sought to reorient the implementation of UNS into a comprehensive, structured package. This included a dedicated training manual, standardised operating procedures with clearly defined roles and responsibilities, documentation forms and formats, monitoring tools, and follow-up mechanisms. Drawing on insights from this pilot implementation, these guidance documents and formats will be refined and updated before scaling up as a national initiative across other institutions.

Overall, the findings of this pilot study demonstrate that the UNS package is feasible and acceptable within the existing health system of Sri Lanka. High screening coverage for congenital hypothyroidism, eye abnormalities, and CCHD underscores the readiness of the healthcare system to incorporate these components nationally. However, the relatively low coverage of hearing screening highlights the need for investments in equipment, workforce training, and maintenance systems to ensure equitable access to all newborn screening services. Future efforts can focus on improving the NBS package by implementing universal transcutaneous bilirubin screening at discharge for newborn hyperbilirubinemia. Large-scale implementation studies and cost-effectiveness analyses will be valuable for guiding the nationwide rollout of the UNS package and for establishing a sustainable model for early detection and management of congenital disorders in Sri Lanka.

## Conclusion

The pilot implementation of the UNS programme in Sri Lanka demonstrated significantly high screening coverage for eye abnormalities, congenital hypothyroidism, and CCHD, with detection sensitivity for congenital hypothyroidism and CCHD comparable to internationally recommended standards. In contrast, screening coverage for hearing impairment remained suboptimal, largely due to logistical and capacity-related constraints. These findings confirm that the UNS package is both feasible and well-integrated into the existing health system of Sri Lanka, especially at the tertiary care level. With strategic investments in infrastructure, maintenance, and workforce training, the country is well-positioned to scale up a comprehensive national newborn screening programme aligned with global health and early childhood development priorities.

## Data Availability

The datasets used and analysed during the current study are available from the corresponding author on reasonable request.

## Abbreviations

CCHD: Critical congenital heart diseases
CHDR: Child health development record
OAE: Otoacoustic emission
TSH: Thyroid Stimulating Hormone
UNS: Universal Newborn Screening
WHO: World Health Organisation
